# Robotic excision of deep infiltrating endometriosis at the uretero-vesical junction

**DOI:** 10.1590/S1677-5538.IBJU.2018.0836

**Published:** 2020-01-13

**Authors:** Nupur Tamhane, Lucas Wiegand, Emad Mikhail

**Affiliations:** 1 Department of Obstetrics and Gynecology, University of South Florida, Morsani College of Medicine, Tampa, FL, USA; 2 Department of Urology, University of South Florida, Morsani College of Medicine, Tampa, FL, USA

## Abstract

**Objective:**

To describe the surgical approach for Deep Infiltrating Endometriosis (DIE) of the uretero-vesical junction and bladder reconstruction.

**Materials and methods:**

Bimodal visualization with the help of cystoscopy and robotic-assisted laparoscopy is a useful technique that can be used to delineate the deep infiltrating endometriotic lesion of the bladder wall.

**Results:**

We present the case of a 36 year old G3P3 woman, with right sided hydroureter/hydronephrosis and biopsy proven DIE. Pre-operative MRI was suggestive of bladder wall lesion involving the posterior right bladder wall and extending to right uretero-vesical junction. On entry into the abdomen, the pelvis looked normal except for the right sided hydroureter. Hysterectomy was performed without difficulty. Bimodal visualization was then utilized to delineate the endometriotic lesion. Cystotomy was then performed and endometriotic lesion of the bladder was subsequently excised. This was followed by right sided ureterolysis and excision of endometriotic lesion of uretero-vesical junction. Bladder was reconstructed and the ureter was re-implanted. Psoas hitch was performed to reduce tension on the anastomosis. Post-operative course was uneventful. Retrograde cystogram performed one month post-operatively showed no contrast leak after re-implantation of right ureter. Foleys catheter was removed at 4 weeks post-operatively. Ureteric stent and nephrostomy tube were removed post-operatively at 8 weeks and 9 weeks respectively.

**Conclusion:**

Deep infiltrating endometriosis can be present in normal looking pelvis. In patients with deep infiltrating endometriosis of the bladder, bimodal visualization might be needed to delineate the extent of the disease.

## ARTICLE INFO

**Available at: http://www.intbrazjurol.com.br/video-section/20180836_Tamhane_et_al**

**Int Braz J Urol. 2020; 46 (Video #3): 140-140**

